# Improving global maternal and newborn survival via innovation: Stakeholder perspectives on the Saving Lives at Birth Grand Challenge

**DOI:** 10.1371/journal.pone.0254589

**Published:** 2021-07-14

**Authors:** Amy Finnegan, Blen Biru, Andrea Taylor, Sowmya Rajan, Krishna Udayakumar, Joy Noel Baumgartner

**Affiliations:** 1 Evidence Lab, Duke Global Health Institute, Duke University, Durham, North Carolina, United States of America; 2 Duke Global Health Innovation Center, Duke Global Health Institute, Duke University, Durham, North Carolina, United States of America; Universiteit Maastricht, NETHERLANDS

## Abstract

The Saving Lives at Birth (SL@B) funding partners joined in 2011 to source, support, and scale maternal and newborn health (MNH) innovations to improve maternal and newborn survival by focusing on the 24 hours around the time of birth. A multi-methods, retrospective portfolio evaluation was conducted to determine SL@B’s impact. Forty semi-structured, key informant interviews (KIIs) were conducted with experts in global MNH based in low- and middle-income and in high-income countries to assess the SL@B program. KIIs were conducted with global MNH technical experts, innovators, government officials in low- and middle-income countries, donors, private investors, and implementing partners to include the full spectrum of voices involved in identifying and scaling innovations. Data were analyzed using thematic analysis. Stakeholders believe the SL@B program has been successful in changing the way maternal and newborn health programs are delivered with a focus on doing things differently through innovation. The open approach to sourcing innovation was seen as positive to the extent that it brought more interdisciplinary stakeholders to think about the problem of maternal and newborn survival. However, a demand-driven approach that aims to source innovations that address MNH priority needs and takes into account the needs of end users (e.g. individuals and governments) was suggested as a strategy for ensuring that more innovations go to scale.

## Introduction

Progress on reducing maternal and newborn mortality has been inadequate. Far too many women and newborns die in the 24 hours around birth, the majority in low- and middle-income country (LMIC) settings [1–3]. There is a growing realization in the maternal and newborn health (MNH) space that achieving equity will require doing things differently through innovative approaches [4]. However, best practices for sourcing, supporting the development of, and scaling innovation in MNH in LMIC settings remain unknown.

### The Saving Lives at Birth (SL@B) program

The Saving Lives at Birth (SL@B) program launched in 2011 as a Grand Challenge for Development recognizing that making progress on the Millennium Development Goals (MDGs) related to maternal and newborn survival would require going beyond traditional approaches and that innovation could help leapfrog toward progress [5]. The SL@B funding consortium is comprised of both public and private funders including the United States Agency for International Development (USAID), Grand Challenges Canada (GCC), the Korean International Cooperation Agency (KOICA), the Norwegian Agency for Development Cooperation (Norad), the Bill & Melinda Gates Foundation (BMGF), and the United Kingdom Department for International Development (DFID or UKAID); the World Bank was an early partner and is now an affiliate group.

The SL@B Grand Challenge was designed to crowd-in innovations from a broad array of innovators and support them to reach integrated impact and scale through funding for science and technology, service delivery, and demand generation at the seed, validation, and transition to scale levels. One unique aspect of the SL@B program has been the creation of a “community of innovators” who can rely on each other for support in developing global MNH innovations. The cornerstone of this effort is the DevelopmentXChange (DevX), an annual event traditionally held at USAID headquarters in Washington, D.C. that attracts SL@B innovators, SL@B finalists, and potential scaling partners for SL@B innovations. Innovators compete for SL@B funding at the DevX event and are evaluated by a diverse review panel of MNH experts and investors in order to screen in the most innovative and investable ideas. Each year, SL@B attracted 500+ applications and selected about 60 finalists who competed for between 10–20 awards. Since 2011, the SL@B program has funded 116 unique innovations in eight rounds including 25 innovations that have received multiple rounds of SL@B funding [6]. These innovations address MNH challenges in different countries, and span different growth stages and types.

### The SL@B portfolio of innovations

SL@B awards were made based on the stage of innovation at three different levels: seed—max of 250K USD up to two years to support development of early stage ideas; validation—max of 250K USD up to two years to test the effectiveness of an innovation, and transition-to-Scale (TTS)–max of two million USD up to four years to support scaling of an innovation [7].

While there were 116 unique innovations, because some innovations were funded multiple times as they moved through stages of development, there were 147 total awards, 13 of these were TTS to support transition to scale but not necessarily full-scale delivery. The vast majority (83%) of SL@B awards went to organizations with headquarters in high-income countries (HICs) and implementing partners in low- and middle-income countries (LMICs). SL@B funded different types of innovations in the portfolio including products/devices/diagnostics (61% of awards), mHealth solutions (11%), drugs and vaccines (12%), and service delivery practices/approaches (16%). Innovation awards targeted causes that led to maternal mortality (38%), neonatal mortality (29%), or both (33%).

### Evaluating SL@B

In 2017, the SL@B funding partners solicited an external evaluation of the progress of SL@B in sourcing, supporting the development of, and scaling MNH innovations. The evaluation conducted by our interdisciplinary team included a broad range of mixed-methods approaches. The larger evaluation focused on understanding whether SL@B, one of the first large-scale programs for funding MNH innovations, had been designed optimally to accelerate progress on improving maternal and newborn survival and providing insights on how future MNH innovation programs might be organized and delivered. A quantitative investigation of scaling pathways was part of the full evaluation [8], but is not a focus of this manuscript. The SL@B design components that potentially could have been optimized were selected from the published theory of change [7] and included the following: how SL@B sourced innovations (an open call for ideas from any organization or type of innovation), who SL@B funded (prioritizing representation of LMIC-based innovators), and what SL@B offered beyond the grant itself (non-financial support). The final report with findings from the overall evaluation that draws from different research methods (portfolio analysis, cost-effectiveness analysis, and mixed-methods research) is available online [8].

This paper is comprised of a deep dive analysis where we present the results of in-depth qualitative interviews with a wide array of stakeholders and end users of innovations including global MNH technical experts, MNH donors and private investors, implementing partners, SL@B innovators, and government representatives in countries where SL@B innovations have been tested and/or implemented. We aim to answer the following questions from stakeholders’ perspectives:

- Has the SL@B program been designed optimally to source, support, and scale MNH innovations?
- What are the major achievements of the SL@B program?
- Are there any areas of unrealized potential of the SL@B program that could inform future investments in MNH innovation programs?

While the questions were framed around the SL@B program, we were also soliciting insights more broadly around how to source and scale MNH innovations for impact.

## Materials and methods

### Study design and data collection

In-depth key informant (KI) interviews were conducted in person and via phone (~1 hour in length) between July 2018 and March 2019. We conducted the KI interviews with individuals purposively selected to represent MNH experts and SL@B innovators based both in HICs and LMICs, as well as MNH funders based in HICs (we did not interview any investors or funders from LMICs) and implementing partners and government officials who may be end users of SL@B innovations. We sought to balance the interviews and perspectives by LMIC and HIC voices. All LMIC KIs were nationals and working in the country they represent, only one HIC national was an American working in an LMIC, and global MNH experts who reside primarily in HICs and represent HIC institutions were classified as HIC KIs. We identified KIs by their leadership positions, authorship on high-impact journal articles in MNH topics, and attendance at DevX, plus we used snowball sampling to identify additional KIs. Phone interviews were conducted with participants based in the U.S., U.K., Canada, Switzerland (WHO), Nepal, and Uganda. In-person interviews were conducted at the DevX event in D.C and other global events. Additional in-person interviews were conducted during visits to SL@B implementation sites in Kenya and Ethiopia by meeting with SL@B innovators, implementation partners, local stakeholders from the USAID mission and, when possible, government officials. These two countries in East Africa were chosen because this region is a focus for SL@B. Specifically, innovators working in the Kenyan market represent about 17% of the SL@B portfolio. Ethiopia was chosen as an illustrative country given that multiple SL@B seed and validation grants were funded in this setting but none of the innovations had won SL@B transition-to-scale funding. No key informants contacted refused to be interviewed.

#### Questionnaire design

**Fig 1:**
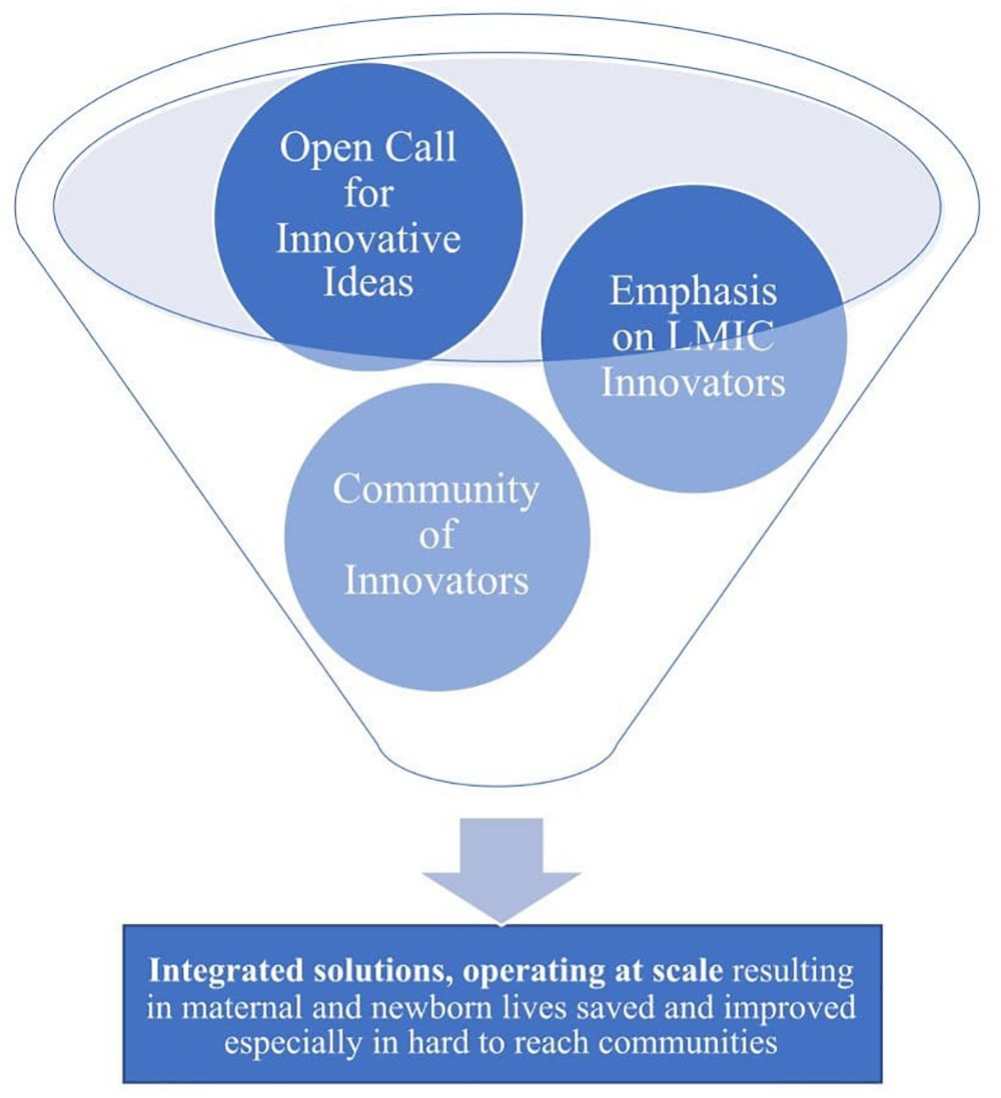
Key components of the SL@B theory of change based on Lalli, et al. (2018).

We conducted interviews using a semi-structured interview guide designed to probe key informant perceptions of the SL@B program’s role in sourcing, supporting the development of, and scaling MNH innovations. Questions were guided by the SL@B theory of change [7] (see Fig 1) including: open call for innovative ideas; emphasis on LMIC-based innovators; and, non-financial support to create a community of innovators and to hold the D.C.-based, annual DevX event.

We also posed open-ended questions about the key achievements and unrealized potential of the SL@B program and solicited recommendations for what SL@B could do better to meet the needs of women and children around the globe. The interviews largely followed the interview guide, but it is important to note that not all questions were asked in all interviews conducted (n = 40) depending on limited knowledge of the KI on certain questions (e.g. government official who has not heard of SL@B program but was a key player in the MNH sector) as well as general interview flow.

#### Data management and analysis

All KI interviews were audiotaped, transcribed, and analyzed by members of the evaluation team. We created a codebook based on the interview guide and refined it based on an initial reading of an interview from each KI group by four members of the research team. Codes were discussed and refined based on this initial reading and additional codes were added. Two authors of this paper (AF & BB) double-coded 10% of the interviews using the NVivo software and met to reconcile any differences. Inter-coder reliability was met at greater than 90% for all codes. The remaining interviews were split roughly in half and coded independently by the same coders (AF & BB).

Transcripts were analyzed using a thematic framework analysis and structured comparison [9]. To achieve the structured comparison, two rounds of memo writing were conducted. In the first round, two co-authors read each coded excerpt stratified by MNH experts (LMICs vs HICs), investors (HICs), and SL@B innovators (LMICs vs HICs) and identified themes. In a second round of memo writing, the emerged themes were aggregated across KI groups when no differences were identified between group responses. Data saturation was confirmed via coding of transcripts and iterative analysis. The team adhered to the consolidated criteria for reporting qualitative research (COREQ) [10] which can be found in the supplementary materials.

### Ethics approval

The Institutional Review Board (IRB) at Duke University approved the study protocols [#2018–0370 and #2018–0617]. All participants provided written informed consent.

## Results

Table 1 shows the perspectives represented by the key informants in the study. The sample was relatively balanced between HIC MNH experts (representing the U.S., U.K., Canada, and Switzerland [WHO]) and MNH experts from LMICs (Kenya, Ethiopia, Uganda, and Nepal) with a slight skew towards LMIC-based voices. Innovators were selected from across the SL@B portfolio to represent the breadth of types of innovation as well as a diverse set of growth stages (seed, validation, and transition to scale), and SL@B funding rounds. Overall, for all thematic domains, the data did not reveal major differences across groups of KIs, unless otherwise noted below, so results are pooled across KI groups.

**Table 1:** Number of key informant perspectives included in the analysis (n = 40).

Key Informants	LMIC- Based	HIC-Based	Total
***Maternal & Neonatal Health (MNH) Experts***	***10***	***11***	***21***
International non-governmental organizations that partner with USAID	3	2	5
USAID leadership (global & missions)	1	5	6
MNH experts (e.g. Ministries of Health, WHO, academics)	6	4	10
***MNH Funders/ Investors***	***-***	***6***	***6***
USAID leadership	-	1	1
Private Sector	-	5	5
***SL@B Innovators***	***9***	***4***	***13***

Note: Eight of the LMIC-based SL@B innovators also provided an MNH expert perspective. SL@B innovation round represented: 8 innovations from rounds 1–4, 5 innovations from rounds 5–8

Innovation type represented: 6 products, 5 practice/approach, 2 drugs/vaccines

SL@B Award type represented: 7 seed, 2 validation, 4 transition to scale (TTS)

### Open call for innovative ideas from organization of any type

Key informants who were familiar with the SL@B program (n = 30 out of 40) were asked about the pros and cons of an open versus targeted approach to identifying and funding innovation, which is a hallmark of the SL@B program. KIs were asked to think about the relative merits of an open versus targeted approach to identifying innovative ideas and funding different types of organizations (e.g. NGOs, for-profits, universities) and innovations.

#### Innovative ideas

Roughly, two-thirds of KIs preferred a targeted approach to soliciting key areas for innovation, where SL@B would drive the innovation agenda on maternal and newborn survival by setting priorities together with LMIC stakeholders. The rest preferred an open approach or had no opinion either way. KIs who were in favor of an open approach thought that this design could bring innovative ideas to the field that would have been impossible to target in advance. KIs who were in favor of a more targeted approach thought that the SL@B platform could be used to focus innovation on priority areas of identified need in maternal and newborn survival. KIs suggested that the needs could be identified in collaboration with LMIC stakeholders: governments, clinicians, civil society organizations, etc. to ensure innovations tackle priority areas and have a higher chance of going to scale.

“*I think we should first have an assessment*, *then identify special critical areas*, *and then focus on [those]*. *Otherwise*, *even if we have innovations*, *we will end up without a significant impact after spending all that time and resources*. *There are areas which we really*, *really need immediate solutions for in newborn and maternal issues*. *So*, *in conclusion*, *I am for a focused approach personally*.”

–LMIC-based MNH expert

#### Type of organization

Overall, most KIs were agnostic about the best type of organization for SL@B to fund (e.g. NGOs, for-profits, and universities). Many commented that no one institution on its own is going to be able to drive change and thus a combination of organizations is needed. Most KIs thought that rather than focusing on the type of organization funded, the innovation should be the focus. If an innovation addresses a critical problem on the ground, is affordable, easy to use, and culturally responsive, then KIs thought that the type of innovator who generated the idea is irrelevant.

“*I think the idea is that innovations should really be recognized that they can come [from anywhere]*. *No one institution or individual has a monopoly on that*, *but it’s just that once you find something that works*, *then how do you make sure the most number of people can benefit from it*? *At that stage is where you really need to make sure you are building on existing systems*, *making sure the right people are involved*, *making sure that people are mobilized around this*. *It basically reaches that critical mass and it’s very difficult to do when it’s just a single initiative driving that forward*.”

–HIC-based MNH expert

A few KIs noted that although universities might have advantages in terms of conducting research and development, they may require more support and guidance when it comes to testing and scaling innovations on the ground.

“*There’s credit in working with universities*, *but again I think it’s facilitating those on the ground connections that are absolutely critical for their successes [which] is sometimes lacking*.”

–HIC-based MNH expert

#### Type of innovation

Across the SL@B portfolio, product-based innovations (diagnostics, and devices) represent 61 percent of all innovations (n = 88). Innovations classified as drugs/ vaccines (n = 16), mHealth (n = 16), and practice and approach (n = 15), are evenly represented, each making up about ten percent of the portfolio overall. When KIs were asked about where SL@B fits in the innovation-funding ecosystem, the majority were keen to point out that SL@B seems to focus on funding devices. However, some KIs expressed concern that devices alone would not be sufficient to improve maternal and newborn survival and that SL@B should be intentional about sourcing integrated solutions that include a wide range of innovations.

One MNH expert observed:

“*But again*, *it depends on the strength of the systems in that case–you could have the best innovation the world*, *but if you don’t have a strong [health] system to implement it—then*, *that’s just not going to work*.”

–HIC-based MNH expert

### Emphasis on LMIC-based innovators

The majority (83%) of awards in SL@B’s portfolio went to organizations based in HICs. KIs were asked to react to this composition and to suggest what could be done to increase LMIC representation, if it were desirable. Almost all KIs (n = 34) weighed in on this question. Two-thirds of KIs emphasized the need to have a more balanced innovation portfolio from the Global South and North. KIs perceived that increased LMIC representation might lead to a more sustainable, cost effective, user friendly, and, most importantly, problem solving innovations in the MNH space.

“*I think there should be a better balance for sure*. *You know*, *people from high-income countries are trying to develop innovations that they think would be appropriate for low-income countries and sometimes they are successful and sometimes they are not*. *People from low-income countries know exactly what the situation is*, *and they know what the possibilities are*, *and what the constraints are; so*, *they might design with those constraints in mind to come up with solutions to work with those constraints*. *So I definitely think that there should be a better balance*.”

–LMIC-based MNH expert

On the other hand, almost a quarter of KIs thought that as long as the innovations that are developed align with needs on the ground, the composition from LMICs is irrelevant.

“*There’s no problem if most of the innovators are from the high-income countries*, *there is no problem*, *but the problem will be—do they really understand the real problem in the low resource setting; that will be a challenge*. *… So to get a solution*, *in general*, *be it from HICs or from LMICs*, *as long as it solves the problem of maternal and child health care*, *there’s no problem*.”

–LMIC-based MNH expert

Many KIs hypothesized that LMIC representation in the SL@B portfolio is low due to lack of awareness of the call for proposals and the competition against HIC innovators who may be more skilled at writing grants, especially in English. To address this gap, several KIs suggested that SL@B should work closely with local ministries, USAID Missions, and LMIC-based NGOs to announce the call. A few KIs suggested that SL@B could source ideas through pre-proposal accelerator workshops or regional events that identify local problems and local innovators, which might include partnerships with HIC innovators and institutions.

### Non-financial support: Community of innovators and the DevelopmentXChange (Devx)

Most KIs thought that the DevX and community of innovators that it has created is one of the major contributions of SL@B. KIs noted that DevX provides a platform like no other that enables innovators to cultivate partnerships that may facilitate market entry and scale, and critical peer-to-peer learning from innovators facing similar challenges (e.g. regulatory hurdles). Most KIs from LMICs thought that SL@B should hold a regional DevX event to enable broader participation from innovators in LMICs who may have trouble obtaining a visa for travel to Washington, D.C., where DevX has traditionally been held.

“*I was amazed to hear the number of innovations going on*. *I even thought*, *before even going to DC [for DevX]*, *we could be having a platform or a forum in Africa where the partners*, *the grantees like us*, *in Africa*, *we just meet in the region to share with one another about what we are doing*.”

–LMIC- based Innovator

### Major achievements of the SL@B program

Key informants were asked their opinion on the major achievements of the SL@B program. Twenty KIs out of the total forty KIs (largely representing the MNH experts group) felt they had enough information to respond to this question. The most frequently mentioned achievement was raising awareness for innovation in maternal and newborn survival.

“*Without SL@B*, *we may not be talking about maternal and newborn survival and innovations; [I think SL@B] tapped attention that would not otherwise have happened*.”

- HIC-based Investor

SL@B was also commended by half of the KIs for encouraging and enabling more ideas, new approaches, and unlike minds to work on maternal and newborn survival, including funders from the public and private sector.

“*I think the fact that anyone is putting innovation up the flagpole and getting some public-private partnerships under way*, *and getting players that typically wouldn’t be at the table to the table to innovate and to join hands in the world of maternal and newborn health is hugely important and a great value add*.”

–HIC-based MNH expert

Finally, a third of KIs specifically mentioned that SL@B has generated a pipeline of innovative ideas that can be taken to scale, which can lead to a broader impact.

“*What SL@B is doing is unique*, *especially by identifying areas that should be taken as priority for MNCH work and then focus on those priority areas*, *and just build a robust pipeline of innovations and innovators that can actually be taken to scale*. *I think where the issue is*, *is about choosing well*, *choosing these interventions well by focusing on those ones that have got the highest propensity to succeed*.”

–LMIC-based MNH expert

KIs were asked to consider their own opinion and describe specific innovations they saw as success stories. Their responses reflected their own interpretation of SL@B’s success which may reflect how much public attention an innovation has received rather than widespread scale. Specific innovations most frequently cited as success stories by KIs included the rapid scale-up of the use of chlorhexidine for umbilical cord care in Nepal [11], which SL@B funded early in its development; Rice University’s NEST 360 program in Malawi, where SL@B has provided seed and transition to scale funding to the Pumani bubble Continuous Positive Airway Pressure (CPAP) device for neonatal respiratory support [12]; and the Every Second Matters Uterine Balloon Tamponade (ESM-UBT) [13] package to treat post-partum hemorrhage, developed by Massachusetts General Hospital.

### Unrealized potential of the SL@B program

While the vast majority of KIs indicated that SL@B has been successful in raising awareness for innovation in maternal and newborn survival, two areas emerged as unrealized potential of the SL@B program. First, many KIs mentioned that, while there have been a few success stories, innovations funded by SL@B often lack scaling plans and have not been integrated into health systems at scale.

KIs hypothesized that lack of scaling success may be due to unclear exit strategies for SL@B-funded innovations, an issue that emerges early in the problem identification and sourcing stage. For example, KIs mentioned the following areas as possible explanations for lack of scale: lack of partnerships with bilateral cooperative agreements for validation and scale, lack of funding partners at later stages identified from the early stages, and a feeling that the current MNH ecosystem is not supportive of innovation, and that the work needed to be done may be beyond the grant-making process and should take a more ecosystem building approach.

A number of KIs expressed concern that SL@B innovations have not reached widespread scale from when the program began (2011) until the time of this study (2019). One participant reflected on the pace of change in MNH through innovation in general:

“*[That innovative new drug] was another one of the very first round awardees*, *wasn’t it*? *So*, *I think it’s just sobering to see how long it takes [to get an innovation to scale]*.”

**–**HIC-based MNH expert

Second, KIs perceived a mismatch between the areas SL@B innovators address and the areas of priority need for MNH innovation in LMICs. KIs expressed their opinion that the innovations funded within the SL@B portfolio do not always address the areas of most need and that the role of SL@B should be, as expressed by one KI, to future-cast the key areas where innovation is needed, by taking a proactive rather than passive approach to the call for innovations so that problems drive the areas for innovation. Several KIs suggested that SL@B could define areas for MNH innovation in collaboration with LMIC partners and design their call for application accordingly.

“*I think the extent of the innovation conversation needs to go further to the frontline… at the end of the day*, *the people who are living with the problem*, *I would say are the best litmus test for the solution*. *I think engaging the global health experts is important*, *but this is not always about expertise*, *it is also about understanding the problem in the context*. … *just creating that ecosystem requires the community from the very beginning*, *not only at the recipient end*, *once you have everything signed off*.”

–LMIC-based MNH expert

## Discussion

According to the KIs interviewed, the SL@B program has successfully generated a pipeline of new, early stage ideas that have the potential to save and improve maternal and neonatal lives [8]. Stakeholders agreed that the SL@B program has ignited interest in the previously neglected innovation space of maternal and newborn survival. Key features of the SL@B program design (open calls and non-financial support) often resonated with stakeholders but there were also suggestions that a targeted, demand-driven call for innovation alongside a renewed emphasis on sourcing more innovations from LMIC-based innovators may help SL@B achieve maximum impact. Likewise, stakeholders discussed that an innovation portfolio that favors innovators from HICs and innovations that do not always align with local, end user, priority MNH areas for innovation may hinder the ability of SL@B-supported innovations to eventually go to scale and reduce maternal and neonatal mortality.

The findings in this study echo the growing literature on the factors that contribute to successfully scaling innovations in LMIC settings [14–21]. Innovations have a higher chance of successfully scaling if they are “relevant for addressing persistent or sharply felt problems” [14] and are consistent with local priorities [21], a sentiment echoed by KIs from across all groups interviewed for this study. Government involvement at the national and/or subnational level in innovation design, implementation, and evaluation is crucial to country ownership—it can ensure that the innovation will align with key priorities on the ground and have a higher chance of reaching widespread scale [16]. Best practices for scaling have been translated into guidance for innovators such as Ready, Set, Launch [22] and repositories like ExpandNet [23] for innovators who seek to adopt best practices for scaling innovations. One study in the pharmaceutical industry found that it can take a drug 17 years to reach widespread use and scale [24]; however, there are no existing studies of when to expect scale from device and practice/approach type innovations in global MNH. It should also be noted that the final evaluation report highlighted that SL@B was most adept at sourcing and supporting early stage innovations; SL@B was not designed for financially backing full scale up nor were the donors necessarily expecting every innovation would scale [8].

The qualitative insights from this evaluation of the SL@B program have implications for future MNH-focused innovation programs. Engaging stakeholders in LMICs before issuing a call for innovative ideas could result in a higher probability that MNH innovations, once tested and proven effective, will be more readily adopted by LMIC decision-makers/end users, integrated into health systems, and reach widespread scale. Innovation funding programs should continue to strive for a balanced portfolio of innovators from LMIC and HIC settings, but the primary foci should still be on whether the innovation itself has the potential for health impact in priority areas, whether it resonates with local end users, and if it is scalable and sustainable within resource-limited LMIC settings. To ensure that the portfolio is balanced, SL@B could consider holding a DevX-like event in LMIC settings (e.g. East Africa) and connect LMIC innovators with pre-application support. Other funders could also adopt a similar strategy to engage local stakeholders and provide additional support to make LMIC innovators more competitive in an application process. One unexpected finding from this evaluation was how little disagreement among stakeholders from different perspectives there was on the key questions of how to balance LMIC and HIC voices in sourcing innovations and on the key role of SL@B in shedding light on innovation as a way to improve MNH.

This qualitative study of the SL@B program has several limitations. The MNH innovation space has grown considerably in the last decade, but we only included SL@B-funded innovators as KIs, and not non-SL@B innovators in the space. Also, this study includes perspectives from government and implementation partners across Kenya, Ethiopia, Uganda, Nepal, and global, high-level experts from HICs, but does not include individuals from every country where SL@B innovations have been deployed. However, within this small sample from areas where SL@B has a large presence, convergence of themes was apparent. This study included 40 interviews which is well over typical benchmarks for qualitative data saturation [25]. One sampling gap is that we have no voices from areas with the highest maternal and newborn disease burdens (e.g. Nigeria, Somalia, and Sudan) where the geographical or political climate may make some of the suggestions in this article not as relevant or applicable. The findings should also be considered in light of the fact that stakeholders were providing subjective responses based on their own experience working in the MNH field as innovators, implementers, donors, and end users and were by no means all experts in scaling innovations though they do represent the broad array of stakeholders involved in sourcing, supporting, and scaling MNH innovations. Questions aimed to understanding the quantifiable impact of SL@B are outside the scope of this manuscript and we refer readers to the full SL@B evaluation [8] for additional insights.

## Conclusion

Eight years of funding innovation through the SL@B program has raised awareness of innovation in MNH and brought new players from the public and private sector to the MNH ecosystem. The open process of sourcing innovative ideas has been driven by innovators rather than identified needs and priorities based on local demand in LMICs. The study highlights the need to make innovation-funding programs more demand-driven so that they provide the most benefit for end users and have a higher likelihood of adoption and wide scale.

## Supporting information

S1 File COREQ checklist. (DOCX)

S2 File MNH influencers interview guide. (PDF)

S3 File SL@B grantee interview guide. (PDF)

S4 File Investor interview guide. (PDF)
