# Supplementary material for: Improving global maternal and newborn survival via innovation: Stakeholder perspectives on the Saving Lives at Birth Grand Challenge
Source: PLoS One. 2021 Jul 14;16(7):e0254589. doi: 10.1371/journal.pone.0254589 (PMC8279342; doi:10.1371/journal.pone.0254589)
Supplement: S1 File — (DOCX) [file pone.0254589.s001.docx]

**COREQ (COnsolidated criteria for REporting Qualitative research) Checklist**

A checklist of items that should be included in reports of qualitative research. You must report the page number in your manuscript where you consider each of the items listed in this checklist. If you have not included this information, either revise your manuscript accordingly before submitting or note N/A.

| **Topic** | **Item No.** | **Guide Questions/Description** | **Reported on Page No.** | **Notes** |
| --- | --- | --- | --- | --- |
| **Domain 1: Research team and reflexivity** | | |  |  |
| *Personal characteristics* | | |  |  |
| Interviewer/facilitator | 1 | Which author/s conducted the interview or focus group? | 2 | AF, BB, JNB, SR and AT conducted the interviews |
| Credentials | 2 | What were the researcher’s credentials? E.g. PhD, MD | Title page | AF=PhD, BB=MSc, JNB=PhD, SR=PhD, AT=MPA, KU= MD, MBA |
| Occupation | 3 | What was their occupation at the time of the study? | N/A | All are researchers affiliated with [XXX] University. |
| Gender | 4 | Was the researcher male or female? | N/A | Five of the researchers are female, one researcher is male. |
| Experience and training | 5 | What experience or training did the researcher have? | 4 | All were trained in research methods. |
| *Relationship with participants* | | |  |  |
| Relationship established | 6 | Was a relationship established prior to study commencement? | 2 | Key informants included experts in MNH and SL@B innovators some of whom the study team were acquainted with and others who were speaking with us for the first time. |
| Participant knowledge of the interviewer | 7 | What did the participants know about the researcher? e.g. personal goals, reasons for doing the research | 2 | Each participants received an introductory email explaining the purpose of the interview and the institution that would be conducting the interview. Each key informant read and signed a consent form explaining key information about the study. |
| Interviewer characteristics | 8 | What characteristics were reported about the interviewer/facilitator? e.g. Bias, assumptions, reasons and interests in the research topic | N/A | The participants were informed that Duke University had been contracted by USAID to conduct an external evaluation of the Saving Lives at Birth (SL@B) program. |
| **Domain 2: Study design** | | |  |  |
| *Theoretical framework* | | |  |  |
| Methodological orientation and Theory | 9 | What methodological orientation was stated to underpin the study? e.g. grounded theory, discourse analysis, ethnography, phenomenology, content analysis | N/A | Content analysis |
| *Participant selection* | | |  |  |
| Sampling | 10 | How were participants selected? e.g. purposive, convenience, consecutive, snowball | 2 | Participants were purposively selected from influential leaders in the maternal and child health field and from the SL@B portfolio of funded innovations. Snowball sampling was used to identify additional respondents. |
| Method of approach | 11 | How were participants approached? e.g. face-to-face, telephone, mail, email | 2 | Each participant received an email from the study team inviting them to participate in the study. |
| Sample size | 12 | How many participants were in the study? | 3 | 40 |
| Non-participation | 13 | How many people refused to participate or dropped out? Reasons? | N/A | None |
| *Setting* | | |  |  |
| Setting of data collection | 14 | Where was the data collected? e.g. home, clinic, workplace | 2 | Data were collected via phone or in-person. Respondents were typically interviewed at their workplace. A few were interviewed in other locations (e.g. coffee shops). |
| Presence of non-participants | 15 | Was anyone else present besides the participants and researchers? | N/A | No. |
| Description of sample | 16 | What are the important characteristics of the sample? e.g. demographic data, date | 3 | Demographic data on respondents was not collected. We collected their background information through the interview and categorized them into groups based on their expertise (innovators, investors, maternal and newborn health experts) and whether they were from a high (HIC) or low- and middle-income country (LMIC). |
| *Data collection* | | |  |  |
| Interview guide | 17 | Were questions, prompts, guides provided by the authors? Was it pilot tested? | 3 | The interview guide was read by the interviewer and participants responded orally. The research team pilot tested the interview guide internally and updated it after a few interviews were conducted with key informants. |
| Repeat interviews | 18 | Were repeat interviews carried out? If yes, how many? | N/A | Interviews were repeated if the participant was not able to complete the interview in the 1 hour time allocated or if technical difficulties during the call led to rescheduling. |
| Audio/visual recording | 19 | Did the research use audio or visual recording to collect the data? | 3 | The interviews were audio recorded with the key informants written consent. |
| Field notes | 20 | Were field notes made during and/or after the interview or focus group? | N/A | Field notes were not systematically recorded. |
| Duration | 21 | What was the duration of the interviews or focus group? | 2 | Each interview lasted approximately 1 hour; at least 3 interviews exceeded 1 hour. |
| Data saturation | 22 | Was data saturation discussed? | 3 | Yes, saturation was confirmed during coding and iterative analysis |
| Transcripts returned | 23 | Were transcripts returned to participants for comment and/or | N/A | Transcripts were not returned to respondents. |
| **Domain 3: analysis and findings** |  |  |  |  |
| *Data analysis* |  |  |  |  |
| Number of coders | 24 | How many data coders coded the data? | 3 | Two coders coded the transcripts. |
| Description of coding tree | 25 | Did authors provide a description of the coding tree? | 3 | The interview guide was used to create the coding tree. |
| Derivation of themes | 26 | Were themes identified in advance or derived from the data? | 3 | Themes were identified in advance from the interview guide with additional emergent themes identified from the data. |
| Software | 27 | What software, if applicable, was used to manage the data? | 3 | NVivo |
| Participant checking | 28 | Did participants provide feedback on the findings? | N/A | No |
| *Reporting* |  |  |  |  |
| Quotations presented | 29 | Were participant quotations presented to illustrate the themes/findings? Was each quotation identified? e.g. participant number | 4,5,6,7,8 | Yes. Quotations were identified based on the KIs expertise. For example, LMIC-based MNH expert, HIC-based MNH expert, LMIC-based innovator, HIC-based innovator, or HIC-based investor. |
| Data and findings consistent | 30 | Was there consistency between the data presented and the findings? | 4-8 | Yes |
| Clarity of major themes | 31 | Were major themes clearly presented in the findings? | 4-8 | Yes |
| Clarity of minor themes | 32 | Is there a description of diverse cases or discussion of minor themes? | 8-9 | Yes |
