# Supplementary material for: Improving global maternal and newborn survival via innovation: Stakeholder perspectives on the Saving Lives at Birth Grand Challenge
Source: PLoS One. 2021 Jul 14;16(7):e0254589. doi: 10.1371/journal.pone.0254589 (PMC8279342; doi:10.1371/journal.pone.0254589)
Supplement: S3 File — (PDF) [file pone.0254589.s003.pdf]

SL@B Grantee Interview Guide

1. **The innovator:** Background information.

Today I want to talk to you about your innovation that was funded by Saving Lives at Birth [*list innovation specific to this innovator*]. We contacted you because you were the primary person listed with SL@B on this grant. If you feel that there is someone else we should talk to about this innovation, please let us know.

First, I would like to start by hearing about your background. Please tell me about your career trajectory. Specifically, have you worked in the MNH space your whole career? If not, what did you do before you changed and what motivated the change?

2. **The Innovation:** What is your innovation?

Now, I would like to hear more about your innovation that was funded by Saving Lives at Birth [*list innovation specific to this innovator*]. What is your innovation and what does it address/solve for?

2A. How long has the organization associated with this innovation been in operation?

2B. Is the innovation still active?

2C. Are there any ways you continue to use the SL@B support?

- Connections
- DevelopmentXChange skills learned
- Accelerator support
- Using the SL@B name when applying for other grants
- Other spin-off organizations that still exist today because of your SL@B funding
- Other?

2D. Did any non-traditional collaborators join you on your SL@B project?

2E. Did your funding help catalyze innovation at your organization? For example, did any of your colleagues choose to apply for SL@B funding or not to apply because of what they saw from your experience?

2F. Are there any new organizations or centers you know of that developed because of SL@B?

2G. Who do you consider the "main players" in the Maternal and Newborn Health (MNH) Ecosystem? By ecosystem, we mean all of the funding sources, implementation agencies, and in-country infrastructure that is the context in which MNH innovations are created and implemented.

- Funders/donors/sponsors
- Implementers
- Government ministries

2H. What do you see as the major gaps in the funding landscape for MNH innovations? Who/what/where isn't getting funded? Where is funding most needed?

3. **The Innovation's/Innovator's Trajectory:**

Now I would like to ask you a few questions to help us understand the trajectory of your innovation. Specifically, I am interested in the trajectory of your innovation from conceiving of the idea, to applying for and receiving funding from SL@B and other sources, and scaling.

3A. How much time elapsed between hearing about SL@B funding and applying for your award?

3B. Before you applied for SL@B funding, was your innovation funded by any other sources, including local funders (internal to your organization, university funds, seed funding)? Please name them and approximate amounts. If you don't feel comfortable sharing the amounts, that's okay.

3C. What would you have done to find support for your innovation if SL@B did not exist? What sources of funding and support would you have targeted?

3D. While you were funded by SL@B, did your innovation receive funding from any others sources, including local/internal funders? Please name them and approximate amounts. If you don't feel comfortable sharing the amounts, that's okay.

3E. What role did the SL@B name and reputation play in obtaining additional funding?

3-optional. ASK ONLY IF AWARD HAS CLOSED: If your SL@B funding has ended, has your innovation received funding from any other sources? Please name them and the approximate amounts. If you don't feel comfortable sharing the amounts, that's okay.

3F. How many times did you apply for SL@B funding before you received your award? If you received multiple, please list for each innovation.

3G. What stage of growth would you classify your innovation in now: idea, prototype, proof-of-concept, scaling, other?

3H. What stage was your innovation in when you were awarded your SL@B award compared to where your innovation is currently: idea, prototype, proof-of-concept, scaling, other?

3I. Do you intend to apply for SL@B funding for this innovation in the future?

IF YES:

3I-yes-a. What level (seed, validation, TTS)?

3I-yes-b. When do you plan to apply?

IF NO:

3I-no-a. Why do you not intend to apply to SL@B for funding for this innovation?

3J. After you received a SL@B award, did you submit a SL@B application in another round that was not selected?

IF YES:

3J-yes-a. Could you share more details about that application/ innovation?

3J-yes-b. What is the status of that innovation now?

IF NO:

3J-no-a. If you didn't submit another application, please explain why not.

3K. Has your innovation been adopted by any country governments or incorporated into any USAID missions?

IF YES:

3K-yes-a. Please describe the process and experience of your innovation being adopted by a country government or USAID.

IF NO:

3K-no-a. Do you intend to do this?
3K-no-b. What is your plan and timeline?

3L. Is there anything else you would like to tell us about the trajectory of your innovation?

4. **The Innovation/Innovator & SL@B:** How was your innovation trajectory impacted by SL@B support?

4A. What support or technical assistance did you receive from SL@B other than funding?

- Technical assistance
- Networking
- Accelerator through VentureWell

4B. Did you feel like you were in a "community of innovators" through the SL@B program?

IF YES:

4B-yes-a.What was the value added of being in a "community of innovators" through the SL@B program?

IF NO:

4B-no-a. Could SL@B have done more to make you feel like you were in a "community of innovators"?

4C. What challenges have you faced and what support did USAID provide to help overcome these obstacles?

4D. Do you think SL@B provided too little, too much or just the right amount of support? Please explain.

4E. Could SL@B have been more helpful in providing support to your organization/ innovation to transition to scale? Please describe.

4F. Are there any resources or pieces of information you wish you would have known <u>before you tried to develop your innovation</u>?

4G. Are there any resources or pieces of information you wish you would have known <u>while you were developing your innovation</u> with SL@B support?

4H. Did your innovation ever receive a no-cost-extension from SL@B?

IF YES:

4H-yes-a. What was the reason you needed a no-cost-extension?

4H-yes-b. What was your experience requesting a no-cost-extension from USAID?

4H-yes-c. Did this help you achieve your goals? Please describe.

4I- Did you ever pivot the innovation during your SL@B grant period?

IF YES:

4I-yes-a. Can you explain the process and your experience of SL@B support?

4J. Is there anything else you'd like to share about your experience with SL@B support?

4K. Does the process of SL@B procurement (e.g. sourcing innovations through an open call for ideas and funding the "best" ideas) meet the needs of the MNH community which you described earlier?

[*PROBE: You listed…]*

4L. SL@B uses an open call for ideas rather than a targeted approach. What do you think are the relative pros and cons of both approaches?

4M. SL@B funds a number of different types of innovators (university, non-profit, for-profit, public international organizations, etc.). What do you think are the relative pros and cons of funding these types of organizations?

- University-based innovators
- Non-profit organizations
- For-profit organizations
- Public international organization (PIO) (e.g. the WHO)

4N. Roughly 80 percent of SL@B innovators are based in high income countries. What do you think of this composition? What could SL@B do to increase the representation of innovators from LMIC in their portfolio, if that's desirable?

4O. What could SL@B do better to improve the lives of women and children across the globe?

4P. If you think SL@B should evolve, in what ways should SL@B evolve?

5. The innovation's Potential Impact

Now we would like to ask a few details specifically about the potential impact of your innovation. These questions will help understand the types of information that is helpful for you as an innovator.

5A. Has there been any economic analysis conducted related to your innovation? Please describe.

IF YES:

5A-yes-a. Did you use your own staff for this analysis or did you bring in additional staff to conduct the analysis?

5A-yes-b. What challenges did you face with performing economic analysis?

IF NO:

5A-no-a. Why has no economic analysis been conducted for your innovation?

5B. What motivated your decision to perform or not perform economic analysis for your innovation?

5C. Have you tried to estimate the costs to scale your innovation?

IF YES:

5C-yes-a. What was the motivation behind estimating these costs?

5C-yes-b. What were some of the challenges you had?

IF NO:

5C-no-a. Why have you not estimated the costs to scale your innovation?

5D. What would be helpful for you to have a tool to estimate costs to scale?
IF YES:

5D-yes-a. Who would you communicate the results with?

IF NO:

5D-no-a. Why not?

5E. How you define “effectiveness” for your innovation?

5F. Have you measured the effectiveness of your innovation?

IF YES:
5F-yes-a. Have you compared the effectiveness of your innovation to other similar products or processes? Please describe.

IF NO:
5F-no-a. Why have you not measured the effectiveness of your innovation?

5G. Have you used any tools to estimate the social impact (ex. lives saved, lives improved, lives touched, DALYs, etc.) of your innovation? Please describe.

5H. When you reported on the indicator of impact you mentioned from your innovation for the final report you submitted to USAID/SL@B, how did you estimate these numbers?

5I. Would it be valuable to you to have a strategy for estimating the lives saved from your innovation?

IF YES:

5I-yes-a. Who would you communicate these results to?

IF NO:

5I-no-a: Why would this not be valuable?

6. **The future of the Innovation/Innovator:**

6A. What are your aspirations for your innovation in the next:

- 3 years
- 5 years
- 10 years

6B. What's your strategy for achieving these goals?

6C. Do you have a plan that's written down? If yes, what is it?

6D. Do you see SL@B support and influence being important to your innovation over the next 3, 5, and 10 years? Please explain.

- 3 years
- 5 years
- 10 years

6E. What are your top three needs to scale your innovation?

1.
2.
3.

6F. How can SL@B help you and your innovation in achieving those scaling goals?

7. **Other people we should talk to?**

7A. Is there anyone else you think we should talk to who would be able to inform us about the support your innovation received from SL@B?

Thank you for taking the time to speak with me today. As a reminder, I have recorded this conversation and will transcribe my notes. We will contact you directly if there is something we would like to quote directly to USAID/SL@B otherwise, we will report general, de-identified themes that have emerged from this interview.
