# Supplementary material for: Improving global maternal and newborn survival via innovation: Stakeholder perspectives on the Saving Lives at Birth Grand Challenge
Source: PLoS One. 2021 Jul 14;16(7):e0254589. doi: 10.1371/journal.pone.0254589 (PMC8279342; doi:10.1371/journal.pone.0254589)
Supplement: S4 File — (PDF) [file pone.0254589.s004.pdf]

MNH Investors Interview Guide

Introduction

Today I want to talk to you about your background and experience in maternal and newborn health and investment funding. I want to hear about your assessment of the most pressing challenges the field faces in achieving reduced MNH mortality and also about the Saving Lives at Birth (SL@B) program.

1. **You & investment in innovation:** What is your background, career trajectory?

1A. First, let's start with a few details about your career. How long have you worked in the MNH investment space? Have you always worked in this space throughout your career? If not, what has your trajectory into this space been?

2. **MNH Challenges:**

2A. In your own assessment, what are the most pressing issues in MNH today and are they the same as those the field faced when you began your career?

2B. Thinking back to 2010 until now, have the most pressing MNH issues changed? In what ways?

3. **Main Players in the MNH Ecosystem:**

3A. Who do you consider the "main players" in the Maternal and Newborn Health (MNH) Ecosystem? By ecosystem, we mean all of the funding sources, implementation agencies, and in-country infrastructure that is the context in which MNH innovations are created and implemented.

- Funders/donors/sponsors
- Implementers
- Government ministries

4. **Evolution of MNH Ecosystem:**

4A. How has the MNH investment ecosystem evolved over the course of time/your career? How about specifically from 2010 until now?

- Funders/donors/sponsors
- Implementers
- Government ministries

4B. Has SL@B support catalyzed investment in global health innovation/innovators? If yes, please describe. If no, where have they fallen short?

4C. What types of investment have been most influential in creating impact in MNH? For example, development impact bonds, blended finance, private capital deployment via traditional or impact investing? Are there any we missed?

4D. Has SL@B support led to self-reliance within countries' own investment sectors through stronger private sectors, empowering people or strengthening core capabilities of countries? If yes, please describe. If no, please elaborate.

4E. Do you think it is now easier or more difficult to fund innovation (i.e. new, untested ideas) in the MNH space than it was prior to 2010? Please explain.

5. **SL@B & the MNH Ecosystem:**

5A. Where does SL@B fit in the MNH investment ecosystem and has that changed over time?

5B. Can you name any SL@B innovations?

5C. What have been the major achievements of SL@B since it began in 2011?

5D. Do you think there has been any unrealized potential of the SL@B program?

5E. What do you think has been SL@B's progress in reducing maternal and newborn mortality and stillbirths?

5F. What specific aspects of the SL@B program have been important? ***[If they do not respond spontaneously about funding, grants management/technical assistance or DevX, ask specifically.]***

- Funding
- Grants management/technical assistance
- The DevelopmentXChange

5G. What are the markers of a successful innovation/new, untested idea in global health?

5H. What is the typical pathway an innovation/new, untested idea takes before it scales up and becomes part of standard practice?
5H-invest. What are some industry standards for scaling up an innovation/new, untested idea in global health?

5I. What are some of the bottlenecks you've seen in regards to funding and scaling innovations/new, untested ideas in MNH?

5J. Do you think that SL@B funding has influenced whether university-based research has become more applied?

6. **SL@B's Role in Sourcing & Scaling MNH Innovations:**

6A. Does the process of SL@B procurement (e.g. sourcing innovations through an open call for ideas and funding the "best" ideas) meet the needs of the MNH community which you described earlier?

6B. SL@B uses an open call for ideas rather than a targeted approach. What do you think are the relative pros and cons of both approaches?

6C. SL@B funds a number of different types of innovators (university, non-profit, for-profit, public international organizations, etc.). What do you think are the relative pros and cons of funding these types of organizations?

- University-based innovators
- Non-profit organizations
- For-profit organizations
- Public international organization (PIO) (e.g. the WHO)

6D. Roughly 80 percent of SL@B innovators are based in high income countries. What do you think of this composition? What could SL@B do to increase the representation of innovators from LMIC in their portfolio, if that's desirable?

6E. What could SL@B do better to improve the lives of women and children across the globe?

7. **Success of MNH:**

7A. What would success look like in the MNH investment field in 3-5 years (2020-2025)?

7B. What would success look like in the MNH investment field in 10 years (2030)?

7C. How can SL@B contribute to that success either in its current form or by evolving?

7D. If you think SL@B should evolve, in what ways should SL@B evolve?

8. **Other people we should talk to?**

8A. Is there anyone else you think we should talk to who would be able to inform us about the investing in global MNH?

Thank you for taking the time to speak with me today. As a reminder, I have recorded this conversation and will transcribe my notes. We will contact you directly if there is something we would like to quote directly to USAID/SL@B otherwise, we will report general, de-identified themes that have emerged from this interview.
